# ﻿Clarifying the phylogenetic placement of Eupoinae Maddison, 2015 (Araneae, Salticidae) with ultra-conserved element data

**DOI:** 10.3897/zookeys.1217.134940

**Published:** 2024-11-12

**Authors:** Junxia Zhang, Yi Ni, Kiran Marathe, Yaozhuo Wang, Wayne P. Maddison

**Affiliations:** 1 Key Laboratory of Zoological Systematics and Application, College of Life Sciences, Hebei University, Baoding, Hebei 071002, China; 2 Hebei Basic Science Center for Biotic Interaction, Hebei University, Baoding, Hebei 071002, China; 3 Department of Zoology and Beaty Biodiversity Museum, University of British Columbia, 6270 University Boulevard, Vancouver, British Columbia, V6T 1Z4, Canada; 4 National Centre for Biological Sciences, Tata Institute of Fundamental Research, GKVK 11 Campus, Bengaluru, 560065, India; 5 Departments of Zoology and Botany and Beaty Biodiversity Museum, University of British Columbia, 6270 University Boulevard, Vancouver, British Columbia, V6T 1Z4, Canada

**Keywords:** Basal lineages, Eupoines, jumping spiders, phylogenomics, UCE

## Abstract

The subfamily Eupoinae Maddison, 2015 is an enigmatic group of minute leaf-litter-dwelling jumping spiders from Southeast Asia. Although previous molecular phylogenetic studies have suggested that it is one of the basal (non-salticine) lineages within jumping spiders, its exact placement remains unclear. In this study, ultra-conserved element data were collected from major salticid lineages to investigate the phylogenetic relationships of all salticid subfamilies, with a special focus on the placement of Eupoinae. The results provide a well-supported phylogeny for jumping spider subfamilies, and suggest a sister relationship of Eupoinae with Spartaeinae Wanless, 1984, a basal lineage of jumping spiders with relatively high species diversity and morphological and behavioural diversity. With the placement of Eupoinae, we have resolved the relationships of all salticid subfamilies, supplying a robust framework for evolutionary studies of jumping spiders.

## ﻿Introduction

**Figures 1, 2.**
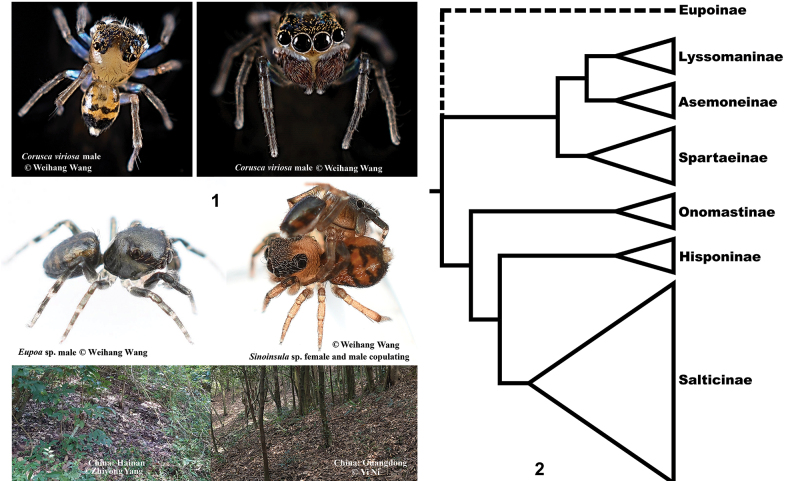
Eupoinae and summary phylogeny of Salticidae**1** photos of living spiders of eupoines and microhabitats **2** summary phylogeny of salticid subfamilies (modified from Maddison et al. 2017).

Salticidae Blackwall, 1841 (jumping spiders) comprises seven subfamilies: Asemoneinae Maddison, 2015, Eupoinae Maddison, 2015, Hisponinae Simon 1901, Lyssomaninae Blackwall, 1841, Onomastinae Maddison, 2015, Spartaeinae Wanless, 1984, and Salticinae Blackwall, 1841 (Maddison 2015). Among them, the subfamily Eupoinae Maddison, 2015 was erected to include an enigmatic group of leaf-litter dwelling jumping spiders known from southern China, Vietnam, Thailand, Malaysia, Laos, and India (Maddison 2015; Fig. 1). Currently 43 species of four genera (*Corusca* Zhou & Li, 2013, *Eupoa* Żabka, 1985, *Megaeupoa* Lin & Li, 2020, and *Sinoinsula* Zhou & Li, 2013) have been described in this subfamily (World Spider Catalog 2024). They are mostly minute spiders, with body size ranging from 1.5 to 5.6 mm, but they possess highly complex genitalic structures, especially the male palps (Żabka 1985; Zhou and Li 2013; Lin and Li 2020).

So far, the phylogenetic relationships of all salticid subfamilies except Eupoinae have been clarified (Maddison et al. 2014, 2017; Fig. 2). Even though previous studies of morphology and molecular phylogeny have suggested that Eupoinae belongs to the basal (non-salticine) lineages of jumping spiders, its placement was unstable in analyses with only a few genes, and thus its exact position on the phylogeny remained uncertain (Maddison et al. 2007, 2014; Maddison 2015). Here we investigate the phylogenetic placement of Eupoinae using ultra-conserved element (UCE) data to fulfill the subfamily-level phylogeny of jumping spiders.

## ﻿Materials and methods

All specimens are preserved in 85–100% ethanol and stored at −20 °C in the Museum of Hebei University, Baoding, China (MHBU) and the Spencer Entomological Collection at the Beaty Biodiversity Museum, University of British Columbia, Vancouver, Canada (UBCZ). The ultra-conserved elements (UCEs) were obtained for 70 taxa that include 68 salticids covering all seven subfamilies and two outgroups (one each of Cheiracanthiidae and Philodromidae). Among them, data were newly collected for 57 taxa in this study, while data for an additional 13 taxa were obtained from previous publications (Zhang et al. 2023; Lin et al. 2024; Yu et al. 2024; Marathe et al. 2024a, 2024b; see Suppl. material 1: table S1 for detailed information). Genomic DNA was extracted using the QIAGEN DNeasy Blood & Tissue Kit. The library preparation was performed with the NEXTFLEX Rapid DNA-Seq Kit 2.0 and the NEXTFLEX Unique Dual Index Barcodes (Set C) (Bioo Scientific) following the protocols described in Zhang et al. (2023). UCE enrichment followed the myBaits protocol 5.01 (Daicel Arbor Biosciences) using a modified version of the RTA probes, the “RTA_v3” probe set (42,213 probes targeting 3818 UCE loci; Zhang et al. 2023). The enriched UCE libraries were then sequenced using the Illumina NovaSeq platform with 150-bp paired-end reads. The UCE loci were extracted from the empirically enriched and sequenced raw reads following the protocols applied in Zhang et al. (2023) with the PHYLUCE (Faircloth 2016) workflow. For seven species with whole genome sequencing data, the genomes were first assembled using the Phylogenomics from Low-coverage Whole-genome Sequencing (PLWS) pipeline (Zhang et al. 2019), and then the UCEs were harvested using the “RTA_v3” probes and the PHYLUCE workflow (see Zhang et al. 2023 for details).

The UCEs extracted from genomes and target enrichment data were combined and organized by locus, and then aligned using Mafft v. 7.313 (Katoh and Standley 2013) with the L-INS-I strategy. Poorly aligned regions were initially trimmed by the heuristic method “-automated1” in trimAl v. 1.4.1 (Capella-Gutiérrez et al. 2009). We then applied Spruceup v. 2020.2.19 (Borowiec 2019) to convert the remaining obviously misaligned fragments to gaps in each alignment (cutoff as 0.7). The gappy regions in each alignment were later masked using Seqtools (PASTA; Mirarab et al. 2014) with “masksites = 35”. An individual gene tree was constructed for each alignment using RAxML v. 8.2.12 (Stamatakis 2014) with the GTRGAMMA model. Gene trees were then inspected using TreeShrink v. 1.3.1 (Mai and Mirarab 2018) to detect and remove sequences that resulted in abnormally long branches on the gene tree. Loci with a length less than 150 bp or less than 50% of taxon occupancy were removed, which resulted in 2685 loci in the final dataset for phylogenetic inference. All remaining UCE loci were concatenated by FASconCAT v. 1.0 (Kück and Meusemann 2010). The maximum-likelihood (ML) analyses were conducted in IQ-TREE v. 2.0.6 (Minh et al. 2020) with the best-fitting model and optimized partition scheme inferred using the option “-m MF+MERGE”. Twenty independent ML tree searches (ten with random starting trees and ten with parsimonious starting trees) were run with the optimized model and partition scheme, and 5000 replicates of ultrafast bootstrap analysis were conducted to assess the node supports. The coalescent-based species-tree method to account for potential gene tree heterogeneity and discordance was also applied. First, the ML tree and 1000 ultrafast bootstrap replicates were inferred in IQ-TREE v. 2.0.6 for each of the remaining UCE loci using the best-fitting model selected by ModelFinder (Kalyaanamoorthy et al. 2017). For each gene tree, the branches with bootstrap ≤ 50% were collapsed by Newick Utils v. 1.6 (Junier and Zdobnov 2010). The Accurate Species Tree Algorithm (ASTRAL-III v. 5.7.1; Zhang et al. 2018) was then applied to estimate the species tree with 100 replicates of bootstrapping to assess the node support.

### ﻿Data resources

The sequenced raw reads were submitted to the GenBank with accession numbers provided in Suppl. material 1: table S1. The alignments of UCE loci, the final concatenated UCE dataset, and the resulting phylogenetic trees are deposited in the Dryad Data Repository at https://doi.org/10.5061/dryad.z08kprrph.

## ﻿Results

**Figure 3.**
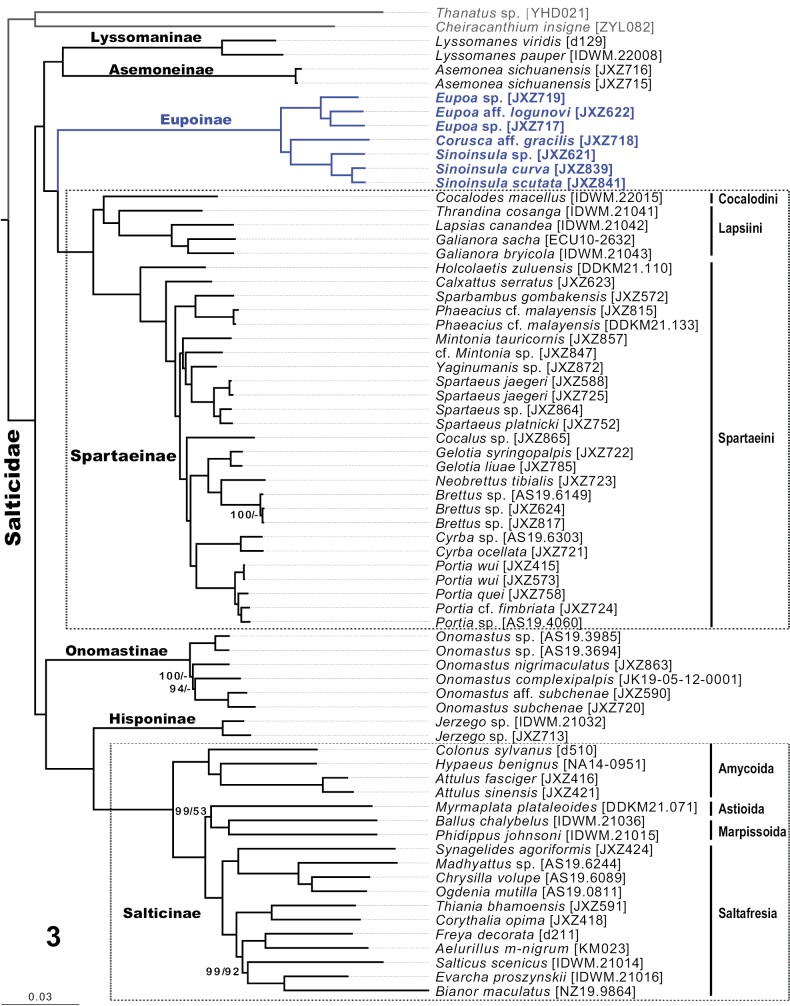
Phylogenetic results. Tree shown is the maximum-likelihood tree from the UCE dataset; numbers along the branches indicate bootstrap support values from the ML (before “/”) and ASTRAL (after “/”) analyses, only numbers lower than 100% are shown; “-” indicates this node is not recovered in the ASTRAL analysis.

The final concatenated dataset of 2685 UCE loci contained 1,109,833 bp and 354,024 parsimony-informative sites. The ML tree is presented in Fig. 3, and the ASTRAL tree is shown in Suppl. material 2: fig. S1. Both results are congruent in the relationships of the salticid subfamilies and strongly support the sister relationship of Eupoinae with Spartaeinae (Fig. 3, Suppl. material 2: fig. S1). Most nodes on the phylogenies gain full support (bootstrap = 100%), with only a few exceptions among the relatively shallower relationships, such as the node with *Salticusscenicus* (Clerck, 1757), *Evarchaproszynskii* Marusik & Logunov, 1998, and *Bianormaculatus* (Keyserling, 1883) (ML bootstrap = 99%, ASTRAL bootstrap = 92%; Fig. 3, Suppl. material 2: fig. S1). The ML and species trees only show minor differences in the relationships of species within *Brettus* Thorell, 1895 and *Onomastus* Simon, 1900 (Fig. 3, Suppl. material 2: fig. S1).

## ﻿Discussion

Since the establishment of the genus *Eupoa* by Żabka (1985), resolving its phylogenetic position has been particularly intriguing due to the unusual male palpal structures observed in this group. Based on two morphological characteristics, the presence of a median apophysis in the male palp and of a tarsal claw in the female palp, *Eupoa* was suggested to be excluded from the clade Salticinae, which contains the bulk of jumping spider diversity (Maddison et al. 2007). Sequences of nuclear and mitochondrial genes (28S, 18S, *wingless*, 16S–ND1, CO1) were obtained for *Eupoanezha* Maddison & Zhang, 2007 to investigate its position on the jumping spider phylogeny (Maddison et al. 2007, 2014). Analyses of these gene regions tended to place *Eupoa* among basal (non-Salticinae) salticids, but they failed to find a clear placement (Maddison et al. 2007, 2014), with *Eupoa* usually outside Salticinae, but occasionally attaching to the long-branched agorines among Salticinae. This may reflect an unusual compositional bias in eupoines (Maddison et al. 2014), or perhaps simply that the limited number of markers that could not resolve the recalcitrant phylogenetic relationships resulting from the rapid radiation of jumping spiders. Later, the genome-wide sequence data were applied to clarify jumping spider phylogeny using the anchored hybrid enrichment (AHE) method. However, no eupoine was included due to a lack of material (Maddison et al. 2017). In this study, seven species from three genera (*Corusca*, *Eupoa*, and *Sinoinsula*) of Eupoinae, along with other major lineages of Salticidae (Suppl. material 1: table S1, Fig. 3), were sampled in the UCE-based phylogenomic analyses. The UCE phylogeny (Fig. 3) recovered the same relationships for the six salticid subfamilies as the AHE result (Maddison et al. 2017; Fig. 2). Unlike previous implications that eupoines may represent a deep-branching lineage long separate from lyssomanines, spartaeines, and other basal groups (Maddison et al. 2007), the UCE phylogenomic results strongly support a sister relationship of Eupoinae with Spartaeinae, which show considerable diversity in morphology and behavior (Wanless 1984; Su et al. 2007).

Although this study did not aim to solve the phylogeny within Eupoinae, the UCE results strongly support a relationship of (*Eupoa* (*Corusca*, *Sinoinsula*)) (Fig. 3, Suppl. material 2: fig. S1). Due to the lack of material, the genus *Megaeupoa* was not sampled in the UCE phylogenomic analyses. Species of *Megaeupoa* also have perplexing male palpal structures like other eupoines, but show significant differences in the somatic characteristics, such as their rather large body size (almost twice as large as species of the other three genera), the presence of a fovea (absent in the other three genera), and the absence of typical eupoine markings (paired paled-colored spots on the abdomen) (Żabka 1985; Maddison et al. 2007; Zhou and Li 2013; Maddison 2015; Lin and Li 2020). Therefore, whether the *Megaeupoa* indeed belongs to Eupoinae or represents an independent lineage in the basal salticids requires further investigation.

It is worth mentioning that although the known species diversity of Eupoinae has dramatically increased in the past decade (Zhou and Li 2013; Logunov and Marusik 2014; Lin and Li 2020; Ying et al. 2021; Wang and Li 2022; Wang et al. 2023; Logunov 2024), we expect that many more species remain to be discovered. Future thorough comparative morphological studies, especially on the genitalic organs, will help to pinpoint the synapomorphies for the clade containing Eupoinae and Spartaeinae, as well as different lineages within Eupoinae.

## ﻿Additional information

### Author ORCIDs

Junxia Zhang https://orcid.org/0000-0003-2179-3954

Yi Ni https://orcid.org/0009-0003-3239-3697

Kiran Marathe https://orcid.org/0000-0002-7364-3475

Yaozhuo Wang https://orcid.org/0009-0007-3252-1838

Wayne P. Maddison https://orcid.org/0000-0003-4953-4575

### Data availability

All of the data that support the findings of this study are available in the main text or Supplementary Information.

## Supplementary materials

Supplementary material 1 Specimen information and summary of harvested UCE loci Data type xlsx https://binary.pensoft.net/file/1173130This dataset is made available under the Open Database License (http://opendatacommons.org/licenses/odbl/1.0/). The Open Database License (ODbL) is a license agreement intended to allow users to freely share, modify, and use this Dataset while maintaining this same freedom for others, provided that the original source and author(s) are credited.Junxia Zhang, Yi Ni, Kiran Marathe, Yaozhuo Wang, Wayne P. Maddison

Supplementary material 2 Phylogenetic result from the ASTRAL analysis Data type pdf Explanation note Numbers along the branches indicate bootstrap support values, only numbers lower than 100% are shown; clades with different relationships from the maximum-likelihood tree are marked in red. https://binary.pensoft.net/file/1173131This dataset is made available under the Open Database License (http://opendatacommons.org/licenses/odbl/1.0/). The Open Database License (ODbL) is a license agreement intended to allow users to freely share, modify, and use this Dataset while maintaining this same freedom for others, provided that the original source and author(s) are credited.Junxia Zhang, Yi Ni, Kiran Marathe, Yaozhuo Wang, Wayne P. Maddison
