# Supplementary material for: ﻿Clarifying the phylogenetic placement of Eupoinae Maddison, 2015 (Araneae, Salticidae) with ultra-conserved element data
Source: Zookeys. 2024 Nov 12;1217:343–51. doi: 10.3897/zookeys.1217.134940 (PMC11576805; doi:10.3897/zookeys.1217.134940)
Supplement: Supplementary material 2 — Phylogenetic result from the ASTRAL analysis [file zookeys-1217-343_article-134940__-s002.pdf]

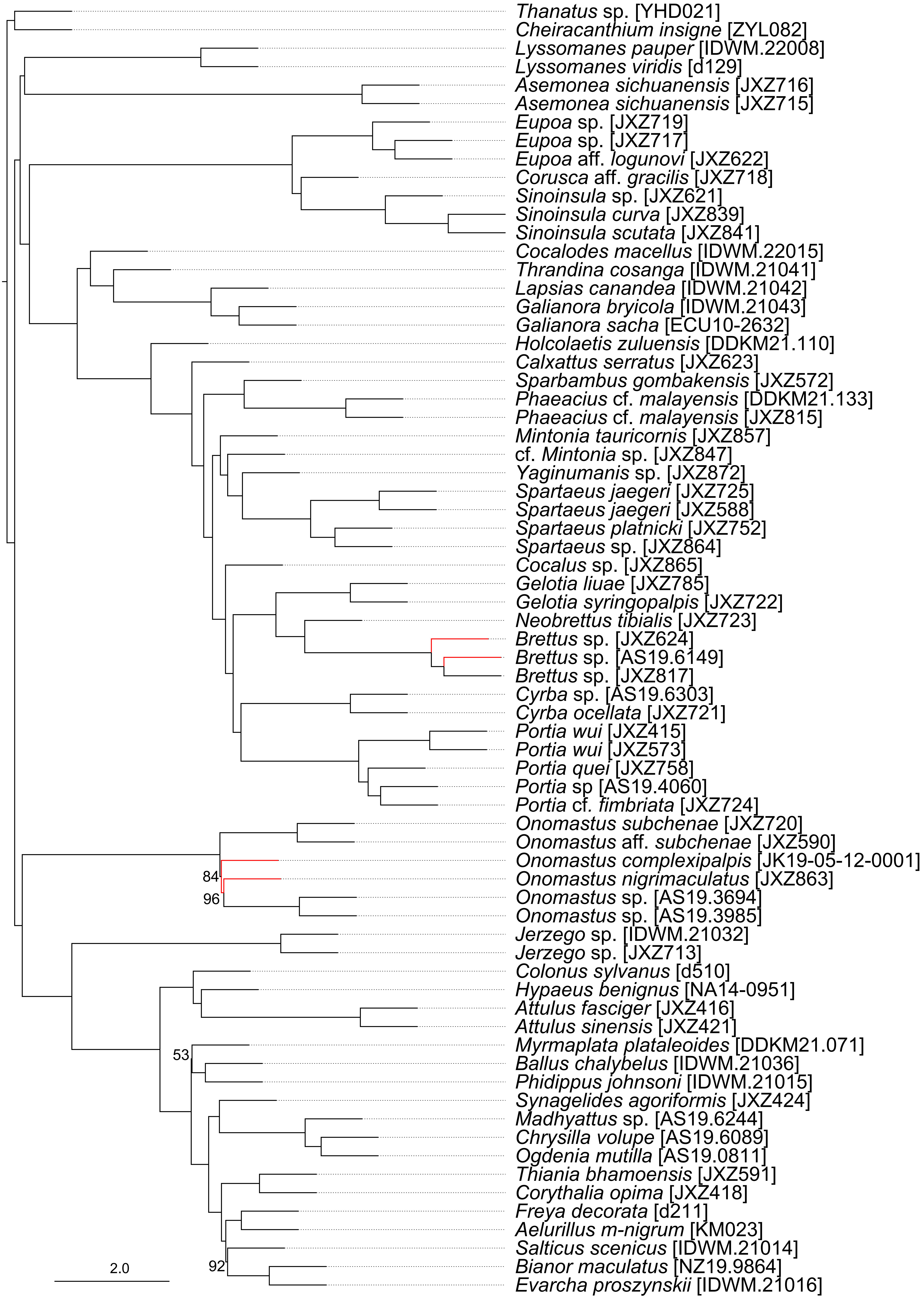

Figure S1. Phylogenetic result from the ASTRAL analysis; numbers along the branches indicate bootstrap support values, only numbers lower than 100% are shown; clades with different relationships from the maximum-likelihood tree are marked in red.
